# Supplementary material for: Sound-driven single-electron transfer in a circuit of coupled quantum rails
Source: Nat Commun. 2019 Oct 8;10:4557. doi: 10.1038/s41467-019-12514-w (PMC6783466; doi:10.1038/s41467-019-12514-w)
Supplement: Supplementary file 2 — Description of Additional Supplementary Files [file 41467_2019_12514_MOESM2_ESM.pdf]

Description of Additional Supplementary Files

File Name: Supplementary Movie 1
Description: Time-dependent simulation of SAW-driven single-electron transfer through the coupling region. Here the peak-to-peak amplitude of the SAW was chosen as 17 meV. At at the beginning of the movie (100 ps) the wave function experiences an abrupt change in the potential landscape causing Landau— Zener transitions into higher energy states. The charge excitation causes an abrupt reduction of the qubit fidelity, F. A small fraction of the wave function escapes the moving QD that is formed by the SAW into neighboring potential minima of the wave train. Tunnel-oscillations are not apparent due to the large number of occupied eigenstates.

File Name: Supplementary Movie 2
Description: Time-dependent simulation of SAW-driven single-electron transfer through the coupling region. Here the peak-to-peak amplitude of the SAW was chosen as 30 meV. The excitation at 100 ps is sufficiently small such that the electron wave function is fully isolated in the moving QD. The charge excitation causes again an abrupt reduction of the qubit fidelity, F. The extent of the excitation is however reduced. Therefore, tunnel oscillations are apparent as the electron propagates along the doublewell potential.

File Name: Supplementary Movie 3
Description: Time-dependent simulation of SAW-driven single-electron transfer through the coupling region. Here the peak-to-peak amplitude of the SAW was chosen as 45 meV. At at the beginning of the movie (100 ps) the electron wave function experiences an abrupt change in the potential landscape. Thanks to the sufficiently strong SAW confinement, excitation into higher energy states is strongly mitigated allowing nearly adiabatic transport. As consequence, the qubit fidelity is only slightly reduced and strong tunnel oscillations occur as the electron propagates along the coupling region.
